# Antibiotic prescribing practices for prophylaxis and therapy of oral/dental infections in pediatric patients – results of a cross-sectional study in Turkey

**DOI:** 10.3205/dgkh000437

**Published:** 2023-05-12

**Authors:** Belen Sirinoglu Capan, Canan Duman, Elif Ece Kalaoglu

**Affiliations:** 1 İstanbul University-Cerrahpasa, Faculty of Dentistry Department of Pediatric Dentistry, İstanbul, Turkey; 2 Istanbul Atlas University, Faculty of Dentistry Department of Pediatric Dentistry, İstanbul, Turkey; 3 Istanbul Gelisim University, Faculty of Dentistry Department of Pediatric Dentistry, İstanbul, Turkey

**Keywords:** antibiotic prescribing practice, pediatric patient, dentists’ knowledge, pedodontists’ knowledge, senior dentistry students’ knowledge, dental education

## Abstract

**Aim:**

Antibiotics are often prescribed for the treatment of various infections and prophylactic purposes in dental practice. Their improper use can cause microbial resistance to antibiotics, which poses a world-wide threat. The aim of this cross-sectional study was to evaluate the knowledge and attitudes of dentists and dentistry students about antibiotic prescription practices for prophylaxis and the treatment of dental infections in pediatric patients.

**Methods:**

A questionnaire was e-mailed to 2,100 dentists and 300 senior dentistry students. The questionnaire was filled out by the participants within a 2-month period (May–June 2020). A 30-point scoring system was developed to assess the knowledge levels of the dentists according to the guidelines. Descriptive statistical analyses were performed. One-way ANOVA test and the Chi-Squared test were used to compare qualitative variables.

**Results:**

The response rate was found to be 24.2% for dentists and 49% for senior dentistry students. 19.4% of the participants were found to be moderately knowledgeable and 80.6% of them were highly knowledgeable. Students’ knowledge scores were found to be higher than the general dentists and other specialists (p<0.05). There was no significant difference between students and pedodontists.

**Conclusion:**

Dentists were found to have sufficient knowledge about the usage of antibiotics in children, but there is still a lack of information about circumstances under which antibiotics should not be prescribed. Dentists and dentistry students should attend continuing education programs to keep their information up-to-date and should also prescribe antibiotics in adherence with the current guidelines to prevent antibiotic resistance.

## Zusammenfassung

**Zielsetzung:**

Antibiotika werden aus prophylaktischer und therapeutischer Indikation in der Zahnarztpraxis verschrieben. Der unsachgemäße Einsatz von Antibiotika kann zu mikrobiellen Resistenzen gegen Antibiotika führen und stellt eine weltweite Bedrohung dar. Ziel der Querschnittsstudie war es, das Wissen und die Einstellungen von Zahnärzten und Zahnmedizinstudenten zur Entscheidung über die Verschreibung von Antibiotika zur Prophylaxe und Behandlung von odontogenen Infektionen bei pädiatrischen Patienten zu evaluieren.

**Methode:**

Per E-Mail wurde ein Fragebogen an 2.100 Zahnärzte und 300 Zahnmedizinstudenten im fortgeschrittenen Studiengang versandt. Der Fragebogen wurde von den Teilnehmern innerhalb von 2 Monaten (Mai–Juni 2020) ausgefüllt. Zur Bewertung des Wissensstandes der Zahnärzte gemäß den Leitlinien wurde ein 30-Punkte-Score-System entwickelt. Deskriptive statistische Analysen wurden durchgeführt. Der Einweg-Anova-Test und der Chi-Quadrat-Test wurden zum Vergleich qualitativer Variablen verwendet.

**Ergebnisse:**

Die Rücklaufquote betrug 24,2% bei Zahnärzten und 49% bei Zahnmedizinstudenten. 19,4% der Teilnehmer wurden als mittelmäßig, 80,6% als sehr gut informiert eingestuft. Die Wissensscore der Studenten waren höher als die der allgemeinen Zahnärzte und anderer Spezialisten (p<0,05). Es gibt keinen signifikanten Unterschied zwischen Studenten und Kinderzahnärzten.

**Schlussfolgerung:**

Es wurde festgestellt, dass Zahnärzte ausreichende Kenntnisse über die Verwendung von Antibiotika bei Kindern haben, aber es gibt immer noch einen Mangel an Informationen, wann Antibiotika nicht verschrieben werden sollten. Zahnärzte und Zahnmedizinstudenten sollten an Fortbildungsprogrammen teilnehmen, um ihre Informationen auf dem neuesten Stand zu halten, und sollten Antibiotika gemäß den aktuellen Richtlinien verschreiben, um Antibiotikaresistenzen vorzubeugen.

## Introduction

Antibiotics are prescribed in dental practice for prophylactic purposes and the treatment of infections. Since many orofacial infections in humans are odontogenic, antibiotics are among the most frequently prescribed drugs by dentists [1]. Antibiotics are often prescribed empirically, which facilitates the development of antibiotic resistance in oral microorganisms [2].

Improper use of antibiotics in pediatric patients has been reported most frequently in ear infections as well as dental infections [2]. Although antibiotic treatment prescribed in orofacial infections can yield very successful results, antibiotics should not be the primary treatment unless there is systemic involvement [3]. Primary treatment should be the dental procedures that affect the main source of infection, such as drainage or pulp treatments.

In recent years, improper (wrong or overdose) use of broad-spectrum antibiotics has caused increased microbial resistance to antibiotics in both children and adults [4]. The improper use of antibiotics also causes side effects, such as allergy development and gastrointestinal problems. For this reason, it is very important for dentists to know the “rational drug use” and put it into clinical practice. Rational drug use is defined as taking medications in accordance with the clinical needs of patients, at appropriate doses, in sufficient time, at the lowest cost to themselves and society [5].

Information on the use of antibiotics in dentistry is taught in undergraduate courses. Dentists may not possess full knowledge of prescribing antibiotics for pediatric patients after graduation, especially if they do not treat children often. In recent years, studies conducted in different countries have shown that dentists have moderate knowledge about antibiotics and tend to prescribe broad-spectrum antibiotics at high doses [6], [7]. However, information about the indication and doses of antibiotics and prophylaxis is updated frequently. In order to prevent the problem of antibiotic resistance, especially in pediatric patients, dentists should follow the current guidelines, attend continuing education programs, and not prescribe antibiotics unless it is absolutely necessary.

Although the literature contains studies evaluating the knowledge and attitudes of dentists and pedodontists about antibiotic prescription, there is no study in this field related to pediatric patients in Turkey. Among the studies in the literature, none compare senior dentistry students to specialized and general dentists. In this respect, our study will be the first to compare four different groups (senior students, general dentists, pedodontists and other specialists) in terms of pediatric patients. 

The aim of this study is to evaluate the knowledge and attitudes of general dentists, pedodontists, other specialist dentists and senior dentistry students about the need for antibiotics, drug preference and rational drug use in prophylaxis and in the treatment of oral/dental infections in pediatric patients. Our first hypothesis is that pedodontists are more knowledgeable than other groups. The second hypothesis is that senior dental students are at least as knowledgeable as non-specialist dentists.

## Methods

This study was approved by the Ethics Committee of Biruni University (2020/39-04) and in full agreement with the World Medical Association Declaration of Helsinki. Participation in this study was completely voluntary and anonymous. The participants who replied to the anonymous questionnaire were considered to have given their consent to participate. 

The 34-item questionnaire was prepared from former published studies [6], [8], [9] with additional parameters regarding antibiotic prescribing and prophylaxis. To assess the use of the newly developed questionnaire in the target population of dentists, the survey was e-mailed to a small number of dentists (25) for review. The survey was modified based on the recommendations from the pilot test and finalized. The questionnaire was organized through Google Forms. 

The sample size required for the study was calculated based on the total number of the dentists (34,245) in Turkey. The minimum sample size was determined to be 244, according to the power calculation for this study. The questionnaire was sent to the e-mail address of 2,100 dentists through the Turkish Dental Association. The sample size required for the study was calculated based on the total number of senior students (368) in Istanbul. The minimum sample size was determined to be 146, according to the power calculation for this study. The questionnaire was sent to a total of 300 senior dental students in Istanbul through an online link via the social media platform WhatsApp. The questionnaire was answered by the participants during a 2-month period (May–June 2020). The survey consisted of two parts (34 items). The first part involved questions related to the demographic characteristics of the participants (7 items). The second part (27 items) queried the knowledge and attitudes of participants about prescribing antibiotics for odontogenic infections and prophylaxis (including questions about oral/dental cases in which they prescribe antibiotics, which antibiotics they prefer, how they determine the dosage, etc.). A 30-point scoring system was developed to assess the knowledge levels of the dentists according to the American Academy of Pediatric Dentistry (AAPD) and American Association of Endodontists (AAE) guidelines and reviews (Table 1 (Tab. 1)) [8], [10], [11], [12], [13]. Total knowledge level was calculated on the basis of each dentists’ response. Each correct response was given a score of ‘1’ and an incorrect answer ‘0’. The total score of the dentists was calculated by adding up the scores, which ranged from 0 to 30 on a Likert Scale. According to this scale, the knowledge levels were categorized as uninformed (0), slightly knowledgeable (1–10), moderately knowledgeable (11–20), and highly knowledgeable (21–30). The study data were obtained by internet e-survey results established by Google Forms.

### Statistical analysis

Statistical analyses were performed using IBM SPSS version 22 (SPSS IBM; Armonk, NY, USA). In the statistical analysis of the study data, in addition to descriptive statistical methods, one-way ANOVA, Tamhane’s T2 test, Student’s T-test, the Chi-Squared test and the Fisher-Freeman-Halton test were used for comparison of qualitative variables. Significance was set at p<0.05.

## Results

### General characteristics

A total of 656 dentists and senior dental students, 202 (30.8%) males, 454 (69.2%) females, aged between 21 and 70, participated in the study between May and June 2020. The response rate was 24.2% for dentists and 49% for senior dentistry students. There were no missing data, as each item in the questionnaire was mandatory. The mean age of dentists was 31.48±9.44. The study examined four groups: pedodontists, students, general dentists and other specialists. The knowledge scores of participants varied between 12 and 30, with a mean of 22.99±3.23 and a median of 23. Participants were divided into two groups according to their scores as moderately (127 participants, 19.4%) and highly knowledgeable (529 participants, 80.6%). The distribution of the general characteristics of participants is given in Table 2 (Tab. 2).

### Clinical conditions and prophylaxis

Nearly half of the dentists (44.5%) reported that they would prescribe antibiotics in the case of acute apical abscess with no systemic involvement. Approximately half of the participants prescribe antibiotics in cases of acute necrosis ulcerative gingivitis (ANUG) (50%) and dental trauma (48.3%). The majority of dentists performed antibiotic prophylaxis in cases of congenital cardiac anomalies (83.8%), prosthetic heart valve (95.7%) and previous infective endocarditis (97.6%). Only 6 (42.8%) of the 14 orthodontists reported that prophylaxis should be administered while placing an orthodontic band (Table 3 (Tab. 3)).

The rate of prescribing antibiotics by general dentists and students in cases of chronic apical periodontitis was found to be statistically significantly higher than among the pedodontists and other specialists (p<0.05). In case of acute apical abscess (with no systemic involvement), students prescribe significantly less antibiotics than do the other groups (p<0.05). In patients with chronic apical abscess, general dentists prescribe more antibiotics than other groups (p<0.05), while students and other specialists mostly prescribe antibiotics in cases with high fever (p<0.05). In cases of trauma, pedodontists and students mostly recommend the use of antibiotics (p<0.05).

In case of heart murmur, the rates of prophylactic antibiotic prescription by pedodontists were statistically significantly lower compared to other groups. Pedodontists and students recommend prophylaxis more often than other groups for patients with uncontrolled diabetes mellitus, intraligamentary anesthesia, orthodontic band placement and endodontic/surgical interventions involving the apex (p<0.05). In patients using immunosuppressive drugs, general dentists give significantly less importance to prophylaxis than do other groups (p<0.05) (Table 3 (Tab. 3)).

### Non-clinical situations

When non-clinical situations are examined, dentists often do not prescribe antibiotics due to patient request (82.5%) and social relations (74.1%). General dentists prescribe antibiotics upon patient request more often than other groups (p<0.05). In patients who had to wait for a long time for a pedodontist appointment, the prescription rate of “other specialists” was significantly higher than in all other groups (p<0.05). This rate was found to be the lowest among pedodontists.

### Antibiotic preference

The majority (63.6%) of the participants treated 0–3 children per day. 73.3% of 131 dentists who care for 5 or more patients a day were pedodontists. Dentists most often prescribe amoxicillin-clavulanic acid (80.6%) orally. Clindamycin (65.9%) is preferred for patients with penicillin allergy. Dentists often prescribe antibiotics for 5–7 days (75.3%) (Table 4 (Tab. 4)). The rate of applying antibiotic treatment for 5–7 days among pedodontists was found to be significantly higher than all other groups (p<0.05). The antibiotic prescription rate of dentists (6–10 per week) who work at state hospitals (13.1%) were statistically significantly higher than dentists at university hospitals (3.9%), private hospitals (2.2%) and private practices (5.7%), (p <0.05).

The vast majority (94.7%) of the participants adjust the antibiotic dose according to the pediatricpatient’s weight and then prognosis (54.3%), age (52.7%), and recommendation of pharmaceutical companies (25.5%) respectively. While prescribing antibiotics, dentists mostly use the internet (67.7%) as a source, then they prefer to consult their colleagues (52.4%), and consult a Vademecum (a drug guide) (50%).

Only 50.9% of dentists follow current guidelines on antibiotic use, and 71% follow current guidelines on antibiotic prophylaxis. The first antibiotic of choice in prophylaxis is amoxicillin (93.9%), and in case of penicillin allergy, the first choice is clindamycin (73.3%). The majority of general dentists (57.7%) and other specialists (62.3%) do not follow the current antibiotic guidelines about pediatric patients (Table 5 (Tab. 5)). When there is extra-oral swelling, 52.9% of the dentists prefer to administer oral antibiotics and 45.9% use parenteral antibiotics.

Dentists working at state hospitals “sometimes” (34.4%) prescribe antibiotics upon patient request. This was statistically significantly higher than among dentists working at university hospitals (11.2%), private hospitals (16.1%) and private practices (18.9%) (p<0.05). According to social relations (like prescription to friends/relatives if they ask), the rate of “sometimes” prescribing antibiotics is higher among state hospital workers compared to other groups (p<0.05).

### Knowledge scores

All dentists were found to be moderately or highly knowledgeable. The knowledge scores of the students and pedodontists were found to be higher than the general dentists and other specialists (p<0.05). There was no significant difference between students and pedodontists. Dentists with 10 years and less of professional experience are more knowledgeable than dentists who have worked for more than 10 years (p<0.05) (Table 6 (Tab. 6)).

The rate of being highly knowledgeable (72.2%) among general dentists was found to be significantly lower than among students (92.6%) and pedodontists (87.3%) (p<0.05). The rate of being highly knowledgeable among those following the current guidelines on prophylaxis and antibiotic use in children was found to be statistically significantly higher than among those who did not (p<0.05).

## Discussion

Dentists should follow current guidelines and need to participate in continuing education programs for the correct diagnosis and treatment of odontogenic infections, as well as rational drug use. In this study, the knowledge levels of dentists and senior dentistry students about the use of antibiotics in pediatric patients were evaluated. According to our results, dentists who are up to date more well-informed about antibiotic prescription. 

According to current guidelines [8], [11], prescription of antibiotics is recommended under the following clinical conditions: infections with signs of systemic involvement, dental trauma (avulsion), and acute periodontal conditions. In contrast, it is not recommended to prescribe antibiotics in chronic cases and cases without systemic involvement (pulpitis, periapical abscess, etc.). In infections such as pulpitis and periodontitis, it is sufficient to intervene only with operative procedures. Within the population studied, even where there was no systemic involvement in acute dental problems, the majority of dentists tend to prescribe antibiotics. This behavior does not conform to current guidelines, but it is similar to other studies in the literature [6], [14]. 

In a study conducted among pedodontists in the United States, 32% prescribed antibiotics in cases of irreversible pulpitis and 39% in cases of chronic apical abscess (with fistula) [15]. These rates were much lower among pedodontists in the present study (11.9% and 1.7%, respectively). Compared to other studies that examined the tendency of general dentists and students to prescribe antibiotics in acute apical periodontitis and irreversible pulpitis cases, the rates reported in this study were lower [9], [16]. In addition, the reason why the students in this study more consistent with the current guidelines may be due to staying abreast of current information and intensive clinical training of students in Turkey.

In the literature, almost all of the dentists participating in studies that investigated conditions requiring prophylaxis performed prophylaxis in patients with congenital cardiac anomalies, previous infective endocarditis [9], [15] and prosthetic heart valves [17], which was similar to the present study. On the other hand, almost half of the dentists in this study do not perform prophylaxis in cases where prophylaxis is recommended, such as uncontrolled diabetes mellitus and orthodontic band placement. This shows that although dentists are more careful with prophylaxis than clinical situations requiring antibiotics, they do not consistently follow current guidelines. Among the treatments performed for prophylaxis, pedodontists and students perform prophylaxis at a higher rate in conjuction with routine infiltrative anesthesia compared to general dentists and other dental specialists. Actually, current guidelines do not recommend prophylaxis in routine infiltrative anesthesia, but given that the patients being treated are children, this result is thought to be due to greater caution on the part of the pedodontist and students.

When prescribing antibiotics, the indication, patient characteristics, workplace, social relations (like demands of their friends/patients), and many other factors play a role. Although the theoretical goal is to prevent the development of resistance through rational use of medicines, dentists all over the world continue to prescribe antibiotics for non-clinical reasons, like requests of their friends/patients or need to delay the treatment due to work overload. Many studies have shown that dentists commonly systemic antibiotics inappropriately [6], [18]. Uncertain diagnosis, insufficient time and inexperience are among the reasons for improper use. Similar to previous studies, in this study, the prescription of antibiotics according to patient request was mostly performed by dentists working in state hospitals, while this rate was lowest in university hospitals [6], [19]. The rate of antibiotic prescription by dentists in England [14] and the USA [15] for reasons of haste or upon the patient's request is considerably lower than this study. These rates are high in Turkey due to overloaded health and oral-care system and patient demands. Additionally, in this study, the rate of antibiotic prescribing by “other specialists” (specialists who are not pedodontists) was found to be highest in pediatric patients who had to wait a long time for a pedodontist appointment. This result can be interpreted as the dental specialists in the developing world of dentistry do not want to treat patients outside their specialty.

Studies in Turkey [17], [19], [20], [21], Croatia [22] and Spain [16] reported that the most common antibiotic prescribed for dental infections was amoxicillin + clavulanic acid. The results of the present study are in line with these other studies. On the other hand, other studies report amoxicillin alone as the first choice of dentists [6], [7], [15], [18]. Although amoxicillin is a good antibiotic for the treatment of dental infections, it is less antimicrobially effective against beta-lactamase producing bacteria. Therefore, it is recommended that amoxicillin + clavulanic acid should be the first choice in dental infections.

For patients with a penicillin allergy, clindamycin has been recommended as the second choice. A study in Jordan reported that in case of penicillin allergy, the first antibiotic preferred by dentists in dental infections was clarithromycin (erythromycin) (77.8%), followed by clindamycin (22.2%) [6]. However, in this study, clindamycin was the first choice of antibiotic by dentists for patients allergic to penicillin. Our findings are in accordance with many other studies in the literature [16], [18], [23]. 

In Jordan, only 29% of dentists prescribe antibiotics for 5–7 days. In this study, similar to other studies, it was shown that dentists frequently prescribe antibiotics for 5–7 days (75.3%) [21], [24]. In the 2019 AAPD guideline, it is recommended that antibiotics should be prescribed for at least 5 days in order to unfold their effectiveness. For means that the majority of the dentists who participated in this study have the correct information.

In this study, the frequency of antibiotics prescription by dentists working in a state hospital was found to be statistically significantly higher than by the other groups. Dentists working in universities and private practice have the lowest antibiotic prescription rate. We assumed that this is due to the large numbers of patients seen in state hospitals in Turkey and the insufficient time for treatment. Dentists at universities and private practice aim to provide ideal treatment by better adherence to the guidelines.

Dentists can use various sources as a guide for prescription. Uysal et al. [25] reported that dentists used most often a Vademecum medication guide as a source when prescribing antibiotics (75%), followed by consulting their colleagues (48%). In our study, the internet was the most frequently consulted source for prescription guidance (67.7%), followed by consulting with colleagues (52.4%). The difference may be due to the younger age of dentists participating in this study and their more effective use of the internet. Inchara et al. [26] emphasized that dentists in India first adjusted the antibiotic dose according to weight and then according to the clinical prognosis of the disease. The results of the present study agree with those of their study.

The World Health Organization (WHO) has recognized that the inappropriate, indiscriminate, and irrational use of antibiotics has lead to antibiotic resistance as a global problem [27]. Therefore, dentists should also be informed about rational drug use and prescribe antibiotics only after the correct diagnosis. Uysal et al. [25] reported that only half of dentists received education on rational drug use. In this study, the proportion of dentists who received education about rational drug use is higher (60%).

 In the present study, the rate of compliance with the current antibiotic guidelines is much higher than that of similar studies in the literature [9], [25]. The knowledge levels of dentists who participated in this study were measured according to the published guidelines [8], [10], [11]. The proportion of dentists with a high level of knowledge was 80.6%, which agrees with the findings of Uysal et al. [25] (82.6%). However, in a study conducted with Norwegian dentists [5], these were found to be moderately knowledgeable. In our study as in the literature [3], [9], pedodontists were more knowledgeable about prescribing antibiotics for children than were general dentists and other specialists. Some other studies suggest that this difference may be due to the lack of information and clinical experience [6], [24].

In a study solely among dental students in Saudi Arabia [28], students had a good level of knowledge, but low awareness about antibiotic guidelines. Similarly, although most of the students in this study were high knowledgeable, only half of them followed current antibiotic guidelines. Nevertheless, this rate of compliance with guidelines and consequently the level of knowledge were higher than those of general dentists and other specialists participating our study. The senior dentistry students who participated in our study were well informed. This result is thought to be due to the extensive clinical experience during dental education in our country and the opportunity to see a large number of cases in clinical practice.

Studies in the literature often compared general dentists and pedodontists or students among themselves in terms of antibiotic knowledge. However, the literature contains no study comparing students, general dentists, pedodontists and other specialists to each other. In this respect, our study is important because it is the first of its kind in the literature. The knowledge scores of the students were higher than that of the general dentists and other specialists, but similar to that of the pedodontists. In line with these results, the first hypothesis of our study confirmed that the pedodontists were more knowledgeable than the other groups. However, the second hypothesis, that students are only as knowledgeable as general dentists, was rejected. This result may be attributed to the fact that students use the internet more often and effectively, and their knowledge has very recently been acquired. Therefore, their knowledge is gained not by memorizing information, but by adhering to up-to-date guidelines.

The main limitation of our study is that we were unable to examine the actual prescription practice of dentists and asked about their approach to clinical situations rather than case questions. Thus we cannot confirm that the responses we received were accurate. Participants may have misreported their antibiotic prescription habits.

## Conclusions

All dentists who participated in this study had moderately or high knowledge about the use of antibiotics in children; none were found to be possess only little knowledgeable. But there is still a lack of information for situations where no antibiotics should be prescribed. Dentists should prescribe antibiotics in adherence with the guidelines to prevent antibiotic resistance. Although undergraduate education seems sufficient in terms of antibiotic knowledge, postgraduate continuing education programs are necessary to keep their knowledge up to date.

## Notes

### Competing interests

The authors declare that they have no competing interests.

### Funding

This research project did not receive any specific grant from funding agencies in the public, commercial, or not-for-profit sectors.

### Acknowledgements 

The authors thank the study participants for their cooperation.

### Data availability statement

The data that support the findings of this study are available from the corresponding author upon reasonable request.

## Figures and Tables

**Table 1:**
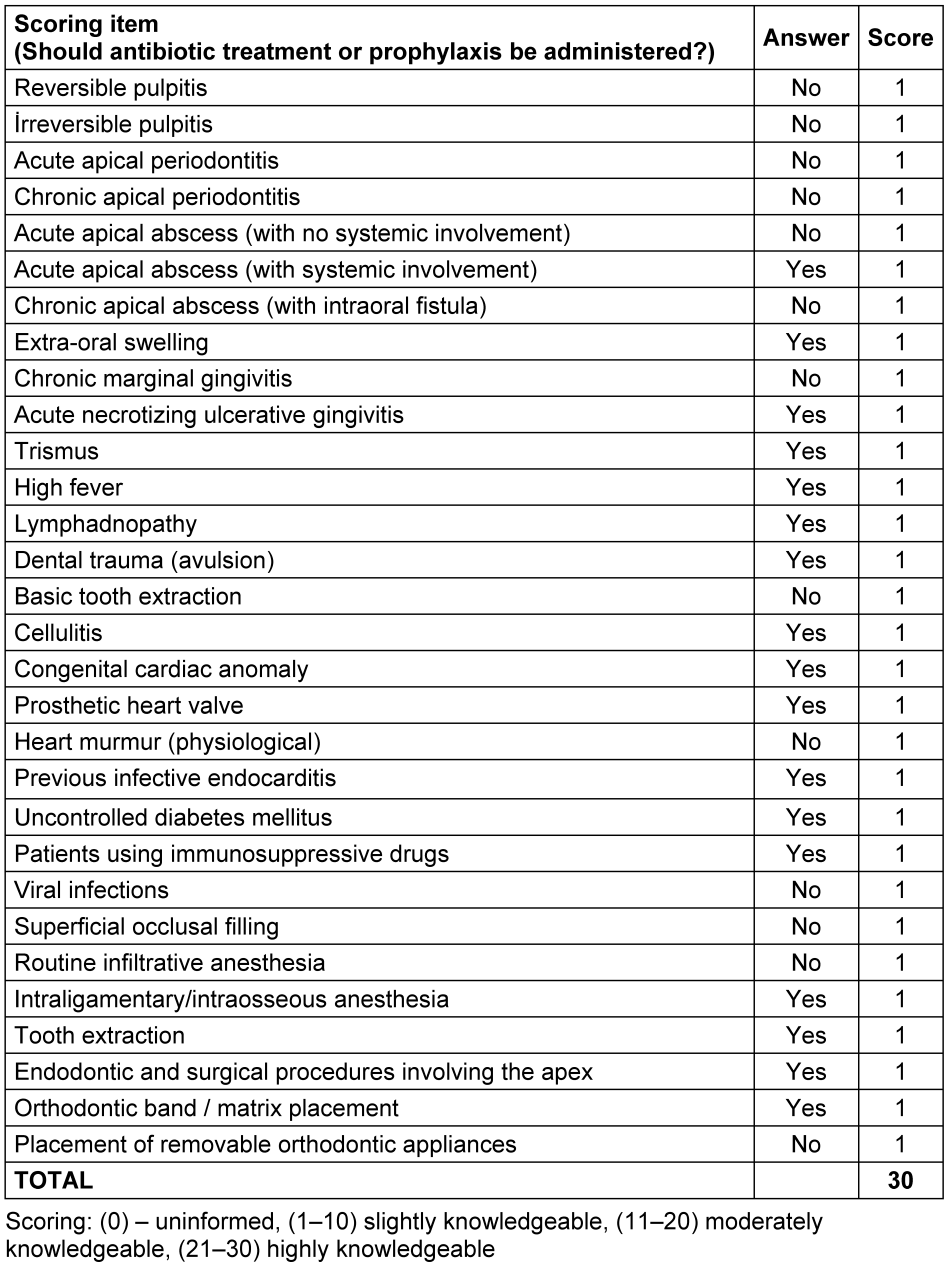
Knowledge level scoring

**Table 2:**
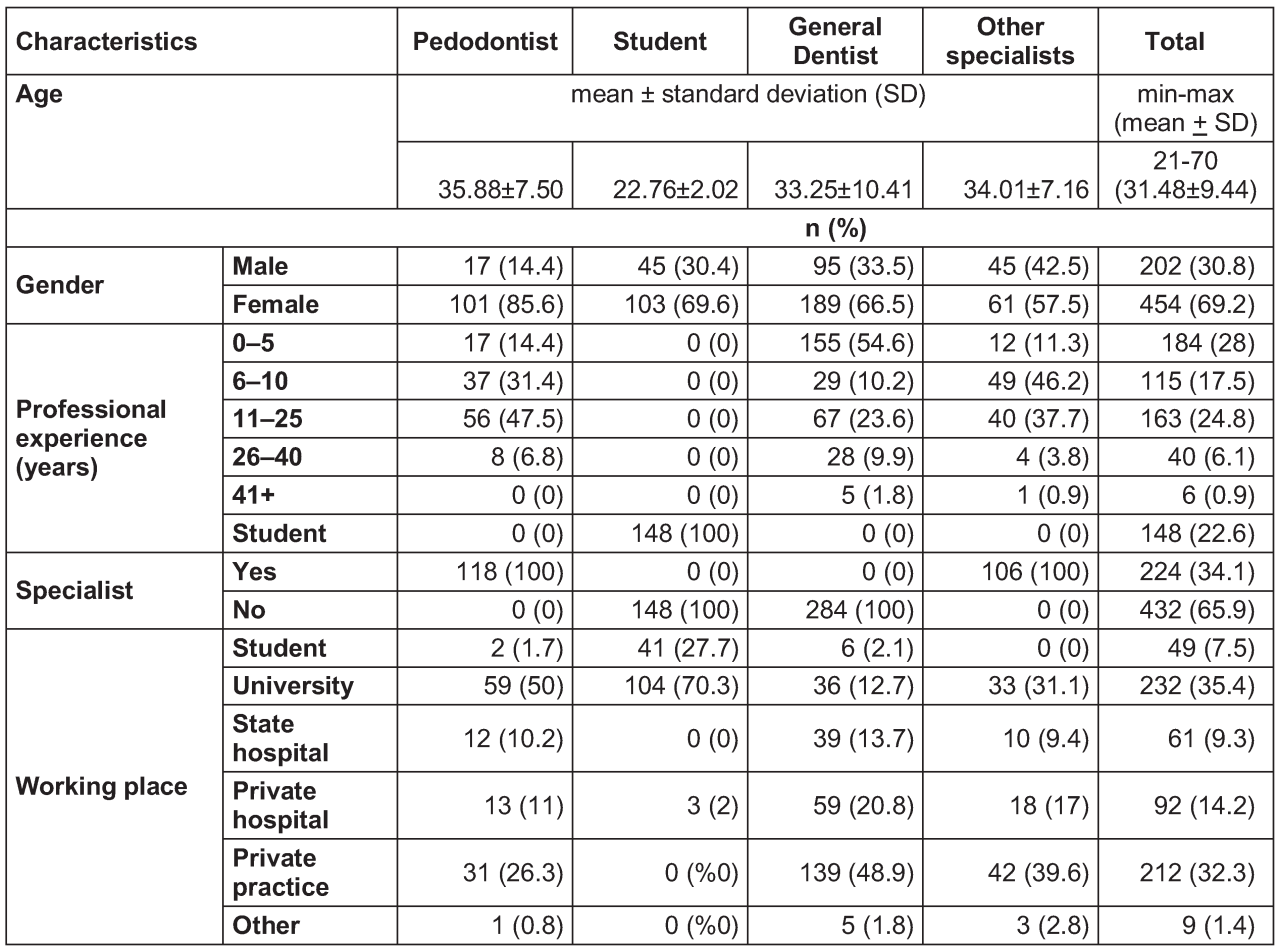
Distribution of the general characteristics of the dentists

**Table 3:**
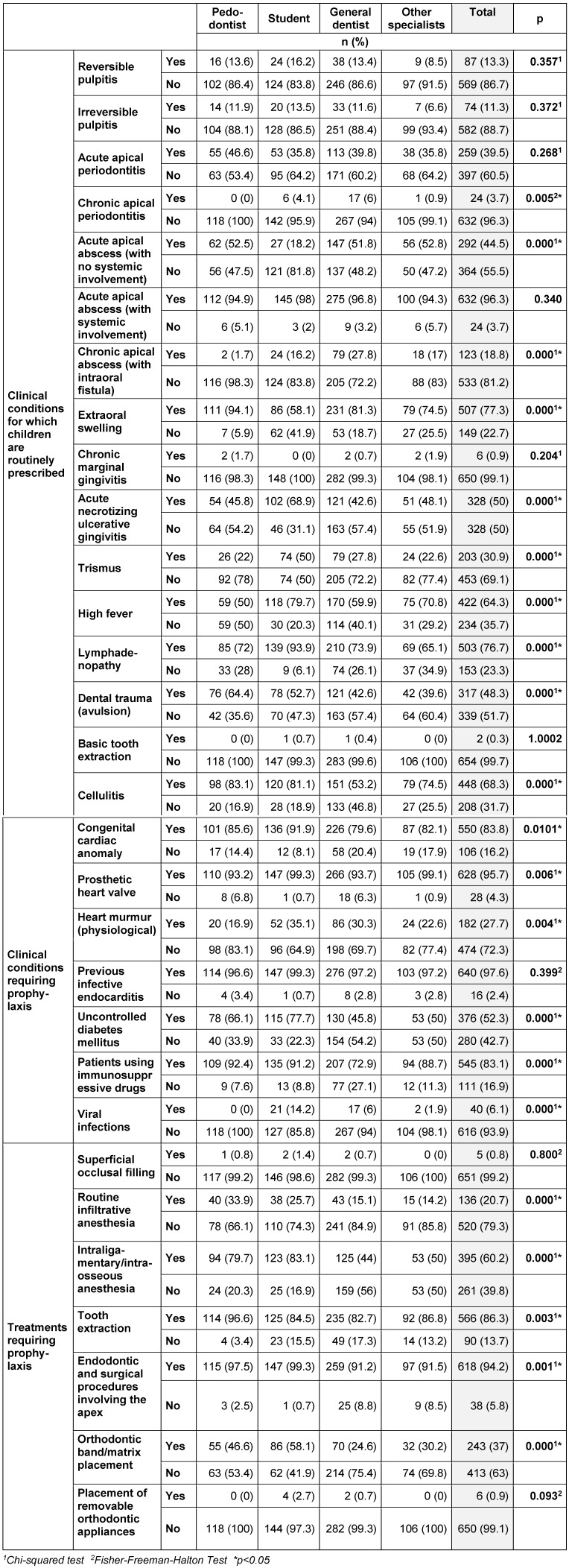
The distribution of clinical conditions and treatments for which children are routinely prescribed antibiotics, including prophylaxis

**Table 4:**
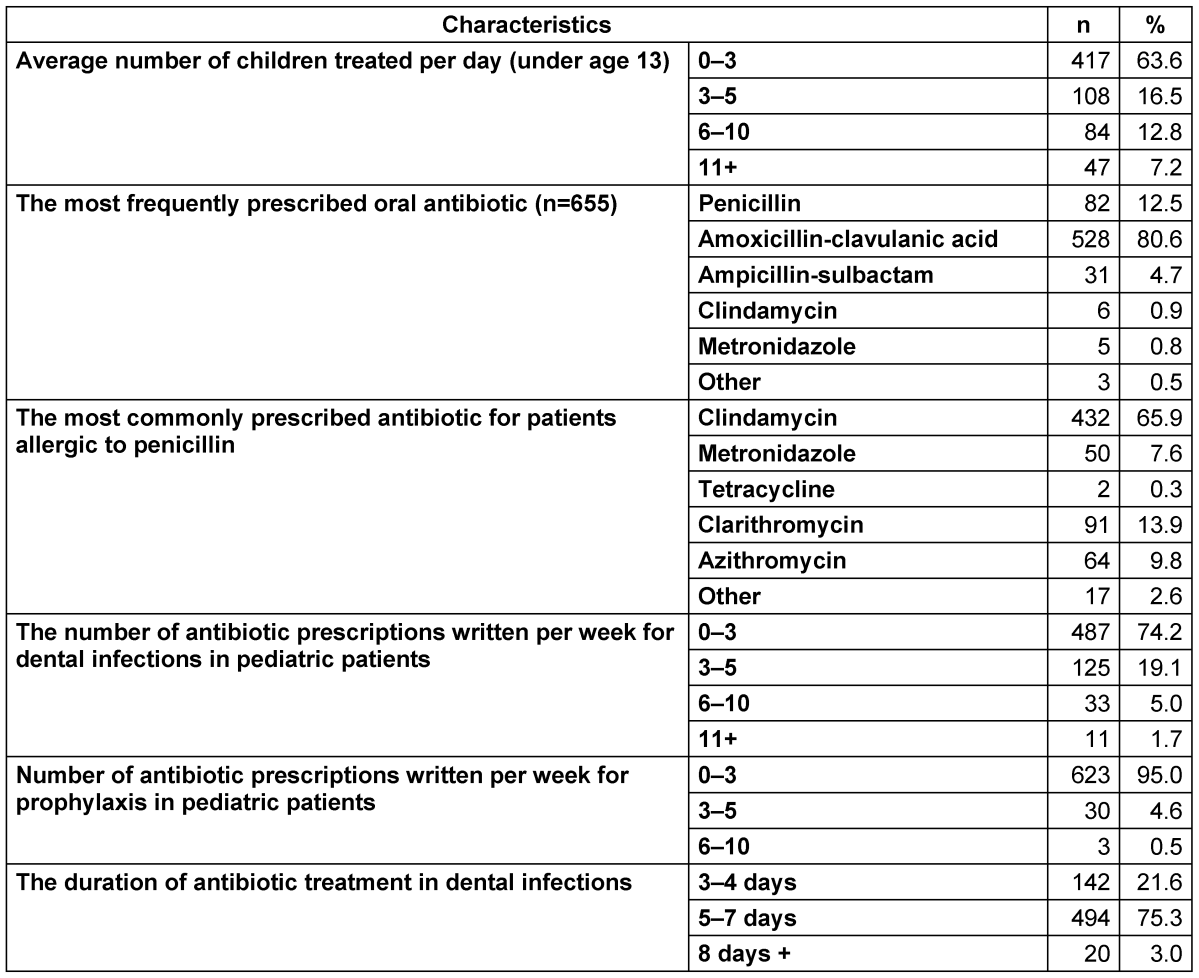
Average number of children treated per day and distribution of data on antibiotic prescriptions

**Table 5:**
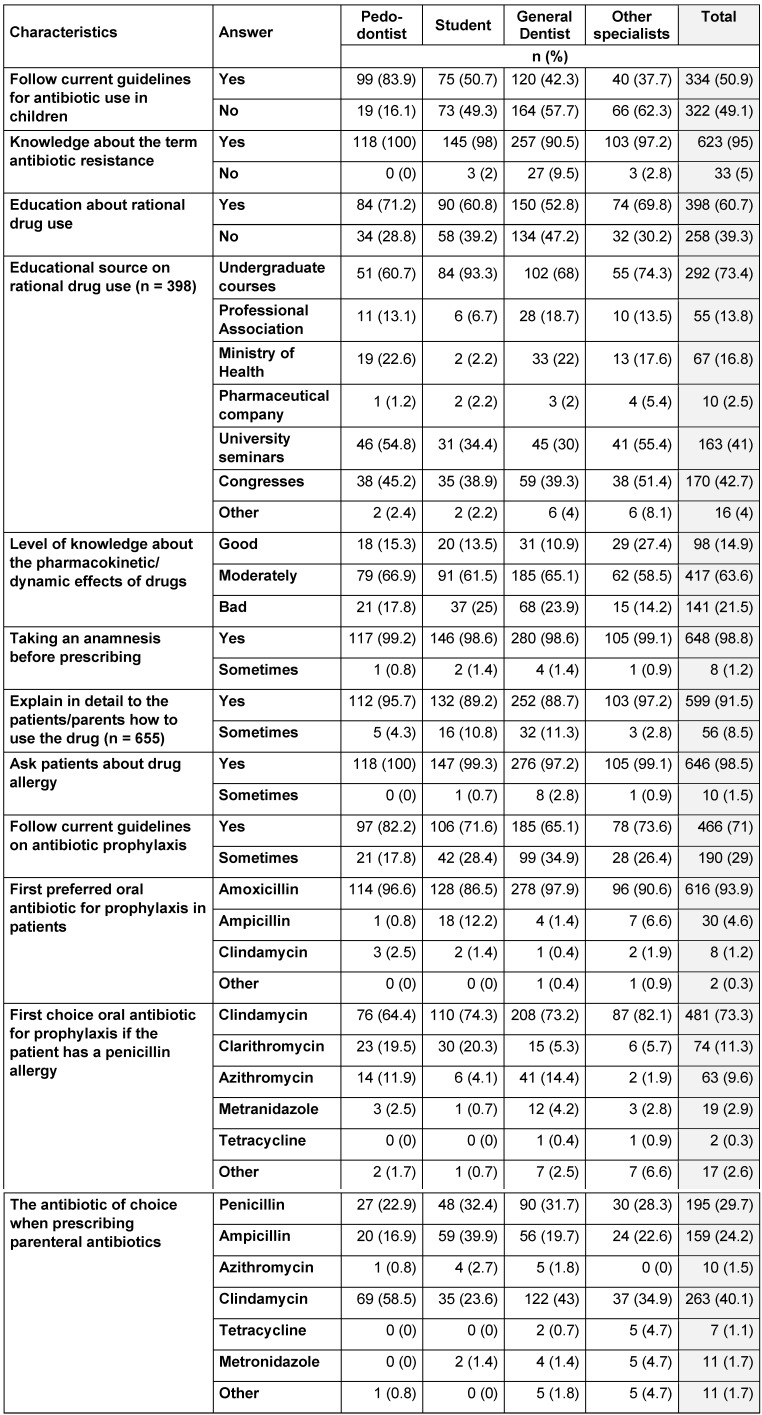
Distribution of data on antibiotic use and rational drug use

**Table 6:**
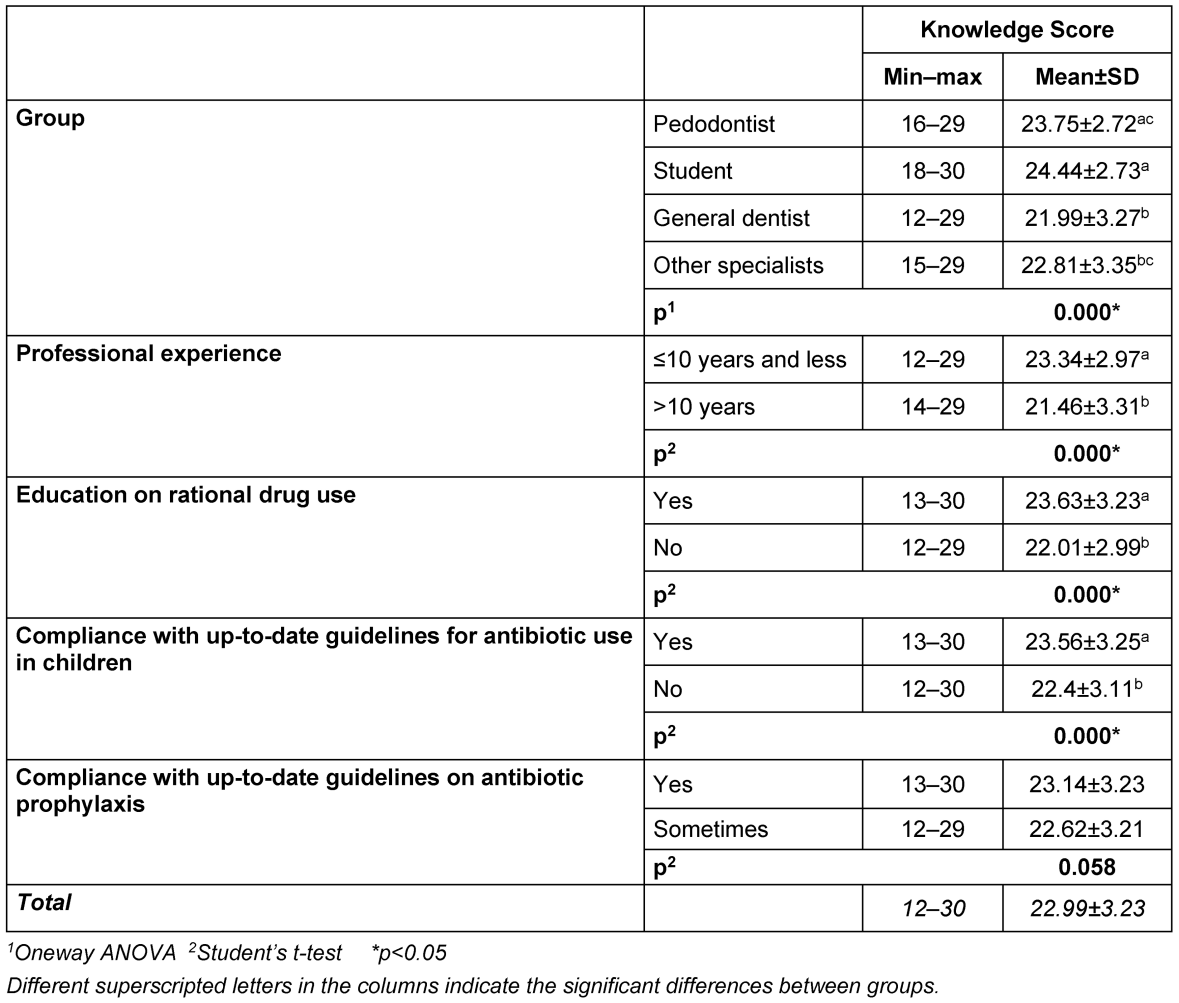
Knowledge score evaluation
